# Proteomic-Based Platelet Activation-Associated Protein SELP May Be a Novel Biomarker for Coagulation and Prognostic in Essential Thrombocythemia

**DOI:** 10.3390/jcm12031078

**Published:** 2023-01-30

**Authors:** Dehao Wang, Pei Zhao, Yan Lv, Jing Ming, Ziqing Wang, Erpeng Yang, Yumeng Li, Mingjing Wang, Jicong Niu, Yanyu Zhang, Yan Sun, Yi Chen, Ke Chen, Zhuo Chen, Weiyi Liu, Xiaomei Hu

**Affiliations:** 1 Graduate School, Beijing University of Chinese Medicine, Beijing 100029, China; 2 Department of Hematology, Xiyuan Hospital, China Academy of Chinese Medical Sciences, Beijing 100091, China; 3 Graduate School, China Academy of Chinese Medical Sciences, Beijing 100700, China; 4 Postdoctoral Research Programme of China Academy of Chinese Medical Sciences, Beijing 100700, China

**Keywords:** essential thrombocythemia, proteomics, SELP, platelet activation, prognostic

## Abstract

Abnormal platelet activation can lead to thrombosis in essential thrombocythemia (ET) and thus impact patient prognosis. Platelet activation-associated proteins are key molecules for platelet activation. However, it is unclear which proteins are most closely associated with the disease’s prognosis. To determine which platelet activation-related proteins can be employed as ET patient prognosis predictors, we used label-free quantification (LFQ) and parallel reaction monitoring (PRM) technology and first determined the serum proteomic expression levels and the differential proteins of ET patients. Then, based on the IPSET (International Prognostic Score for ET), the differential protein associated with the prognostic score was found. To investigate potential processes affecting prognosis, the connection of this protein with prognostic markers, such as thrombotic history, age, white blood cell count, coagulation factors, and inflammatory factors, were further examined. The levels of platelet activation-related proteins GPIbα, SELP, PF4, MMP1, and FLNA were significantly higher in ET patients, according to LFQ and PRM analyses (*p* < 0.01). Based on regression analysis of the IPSET prognostic score, it is suggested that the SELP level was positively correlated with the prognostic score and prognostic risk factor analysis (*p* < 0.05). Further regression analysis of SELP with coagulation factors showed that antithrombin (AT-III) was negatively correlated with SELP levels (*p* < 0.05). Further regression analysis of the inflammatory factors with AT-III and SELP revealed that IL-10, IL-12P70, and IL-31 were negatively correlated with AT-III and SELP (*p* < 0.01). Platelet activation pathway-related proteins are expressed more frequently in ET patients, and serum SELP may be a prognostic marker for these individuals by encouraging leukocyte increase and inflammatory factor expression and causing aberrant coagulation.

## 1. Introduction

The most prevalent form of the chronic myeloproliferative disease, essential thrombocythemia (ET), has a prognosis that depends on several variables, including thrombosis, transleukemia, and fibrosis, the former of which is most common in ET patients and significantly lowers patient survival [1]. The IPSET prognostic model scoring system, which contains three main factors—age, white blood cell count, and history of thrombosis—has also shown good clinical validation in its predictive effect [2]. According to studies, a patient’s chronic inflammatory state is encouraged by advanced age and is also linked to an increase in white blood cell count. A vital function for inflammation in thrombosis, transfibrosis, and leukemia, with the most pronounced encouragement of thrombosis, is played by activated leukocyte proliferation while encouraging the release of inflammatory factors [3]. This has a significant effect on patient prognosis [3]. Thrombotic history mainly reflects the coagulation abnormalities of patients and is most associated with patient survival. Based on the aforementioned investigations, we conclude that the prognostic model’s ability to reflect patients’ coagulation disorders is the key factor contributing to its ability to forecast patient prognosis. Therefore, we tried to find indicators that could be associated with both inflammation and coagulation abnormalities in patients to predict their prognosis.

A common trait of ET patients is the emergence of thrombotic events [4,5]. Activated platelets release both platelet-activating proteins and inflammatory factors, which promote the activation and proliferation of leukocytes [6] and further contribute to the development of chronic inflammation in patients [7]. Few studies have been undertaken on the correlation between platelet activation-related protein expression levels and ET prognosis, despite recent attempts by some researchers to use platelet activation protein expression levels to predict the occurrence of thrombotic events [8]. SELP, also known as P-selectin and CD62P, is one of the key proteins in the platelet activation pathway. It can promote platelet activation, aggregation, and thrombosis, and mediates the adhesion of activated platelets and leukocytes to endothelial cells in the vessel wall, thus affecting the quality of patient survival [9].

Based on these studies, we believe that platelet activation protein expression may be important for patient prognosis. To understand the potential mechanism by which this protein predicts ET prognosis, we, therefore, attempted to identify the most pertinent platelet activation protein for patient prognosis scores using a variety of techniques, such as proteomics. We also further analyzed the relationship between the related protein and prognostic, inflammatory, and coagulation factors.

## 2. Materials and Methods

### 2.1. Study Population from September 2019 to October 2021

Sixty patients with ET who met the inclusion criteria and attended the Xiyuan Hospital outpatient clinic were chosen. Firstly, 22 were randomly chosen for LFQ proteomics testing, while 15 healthy individuals were also included. Then, we randomly selected 14 patients from 60 ET patients and 14 healthy individuals as controls and validated the LFQ proteomics results using parallel reaction monitoring technique (PRM). The PRM verification results were applied to the subsequent correlation analysis. Patients were rediagnosed and reclassified using the 2016 World Health Organization (WHO) [10] diagnostic and classification criteria at the time of their first visit to our institution.

### 2.2. Inclusion Criteria

(1) Met the diagnostic criteria for ET; (2) without regard to gender, aged 18 or older (including 18 years), 85 years or younger (including 85 years); (3) voluntary and duly acknowledged consent; (4) did not participate in other clinical studies.

### 2.3. Exclusion Criteria

(1) People who have experienced severe thrombotic events, such as post-cerebral infarction, consciousness impairment, post-acute myocardial infarction, and severe cardiac insufficiency; those who are affected by serious bleeding, such as intracranial bleeding, gastrointestinal, and urinary bleeding, and those with bleeding from multiple sites throughout the body that produce mild or above anemia) or pulmonary embolism (Killip classification 2, caused by dislodged arterial thrombus in the extremities, and visceral venous thrombosis urgently requiring surgical treatment; (2) those who have used ruxolitinib within the last 4 weeks; (3) patients whose acute leukemia has developed from post-ET myelofibrosis (post-ET MF).

### 2.4. Clinical Data Definition

Definition of prognostic risk factors: variables in the International Prognostic Score for ET (IPSET) system [11] according to WHO 2012—age, white blood cell count, and thrombotic history. Previous thrombotic events were defined as arterial or venous thrombosis, including macrovascular thrombosis and microvascular thrombosis [12], and thrombotic events in this study were primarily judged based on previous case data.

### 2.5. Reagents and Instruments

Protein enrichment volume reagent (Bio-Rad Laboratories, Hercules, CA, USA); 2-D Quant kit (GE Healthcare, Chicago, IL, USA); protein markers (Thermo Fisher Scientific, Waltham, MA, USA). Samples were separated on a high performance liquid chromatograph Orbitrap ExplorisTM 480 (Thermo Fisher Scientific) with system EASY-nLC 1000 (Thermo Fisher Scientific). Nanoliter analytical column (Thermo Fisher Scientific), model 164568; microplate reader enzyme labeler (Hercules, CA, USA), model IMARK.

### 2.6. Serum Specimen Collection

We collected an appropriate amount of fresh blood from the vein of the fasting ET patient using a vacuum blood collection tube containing procoagulant. We let the blood stand at room temperature for 20 min, centrifuged it for 20 min at 3000× *g*, and then separated the serum right away into a −80 °C refrigerator for storage. Blood and serum collection should be completed within 2 h, and specimens should not be repeatedly frozen and thawed.

### 2.7. Protein Lysis, Quantification, and Enzymatic Digestion

Supernatants were transferred to fresh centrifuge tubes after samples were taken at −80 °C and centrifuged at 12,000× *g* for 10 min to eliminate cell debris. Following the manufacturer’s instructions, we removed proteins with a high abundance using the PierceTM Top 14 Abundant Protein Depletion Spin Columns Kit (Thermo Fisher Scientific), then used the BCA Kit to measure protein concentration. Equal amounts of each sample protein were taken for enzymatic digestion and adjusted to the same volume as the lysate. The proteins were then reduced at 56 °C for 30 min with the addition of dithiothreitol (DTT) to achieve a final concentration of 5 mmol/L iodoacetamide (IAA); a final concentration of 11 mmol/L was added and incubated in light at room temperature for 15 min. The sintered samples were put into ultrafiltration tubes, centrifuged for 20 min at 12,000× *g* while it was still room temperature, replaced 3 times with 8 mol/L urea, and then the buffer was replaced 3 times. Trypsin was added at a ratio of 1:50 (protease: protein, m/m) and enzymatically digested overnight. The peptides were recovered by centrifugation at 12,000× *g* for 10 min at room temperature using ultrapure water once and by combining the peptide solutions twice [13].

### 2.8. Liquid Chromatography–Tandem Mass Spectrometry Analysis of Enzymatic Digestion Products

The peptides were separated using an ultra-high-performance liquid system, and then they were introduced into the NSI ion source for ionization and analyzed using Orbitrap ExplorisTM 480 mass spectrometry. The ion source voltage was set to 2.3 kV, and the FAIMS compensation voltage was set to −45 and −70 V. Using a high-resolution Orbitrap, the peptide parent ions and their secondary fragments were found and examined. The primary mass spectrometry scan range was set to 400–1200 *m/z*, and the scan resolution was set to 60,000. TurboTax was set to none, the secondary mass spectrometry resolution was set to 30,000, and the secondary mass spectrometry scan range was fixed at 110 *m/z*. The data acquisition method used was the data-dependent scanning (DDA) procedure, i.e., after a primary scan, the top 15 ions with the highest signal intensity were selected as peptide parental ions after they were sequentially introduced into the HCD collision cell and given fragmentation energy of 27, and then sequentially analyzed by secondary mass spectrometry. To improve the effective utilization of ms, the automatic gain control was set to 300%, the signal width was set to 2E4ions/s, the maximum injection duration was set to 50 ms, and the dynamic exclusion time of the tandem ms scan was set to 30 s to prevent repetitive parent ion scans and increase the effective use of ms [14].

### 2.9. Label-Free Quantification and Bioinformatics Analysis

Proteome Discoverer [Thermo (V2.4.1.15)] was used to retrieve the secondary mass spectrometry data for this work using the homo_Sapiens_9606_PR_20201214.FASTA. The obtained differential proteins were annotated with information based on Uniprot. GO annotation was performed using gene ontology (GO). Metabolic pathway enrichment analysis of the differential proteins was performed using the Kyoto Encyclopedia of Genes and Genomes (KEGG). Using the Wolf Port program, subcellular localization predictions of retrieved proteins were annotated.

### 2.10. PRM Validation

The protein extraction and trypsin digestion of the samples were performed as above. The peptide fragments were separated using an ultra-high-performance liquid system, and then they were injected into an NSI ion source for ionization and onto a Q ExactiveM Plus mass spectrometer for analysis using liquid chromatography–mass spectrometry (LC–MS). The ion source voltage was set at 2.1 kV, and the peptide parent ions and their secondary fragments were detected and analyzed using high resolution Orbitrap. Finally, primary and secondary mass spectrometry scans were performed [15].

### 2.11. Statistical Analysis

SPSS 20.0 software (IBM, Armonk, NY, USA) was used for statistical analysis. A *t*-test was employed to compare the measurement data between the groups. The measurement data were expressed as X ± SD. *p* values obtained from enrichment analysis (Fisher’s exact test) were expressed as bubble plots for functional classification and pathways of significantly enriched differential proteins (*p* < 0.05). A *t*-test *p* < 0.05 for proteins with a 1.3-fold increase or reduction in the factor of differential protein abundance as the difference between protein groups was considered to indicate differential protein abundance. PRM protein validation data were processed using Skyline 20.2. Simple linear regression was used to examine the association between the measures, and statistical differences were taken into account at *p* < 0.05.

## 3. Results

**Figure 1:**
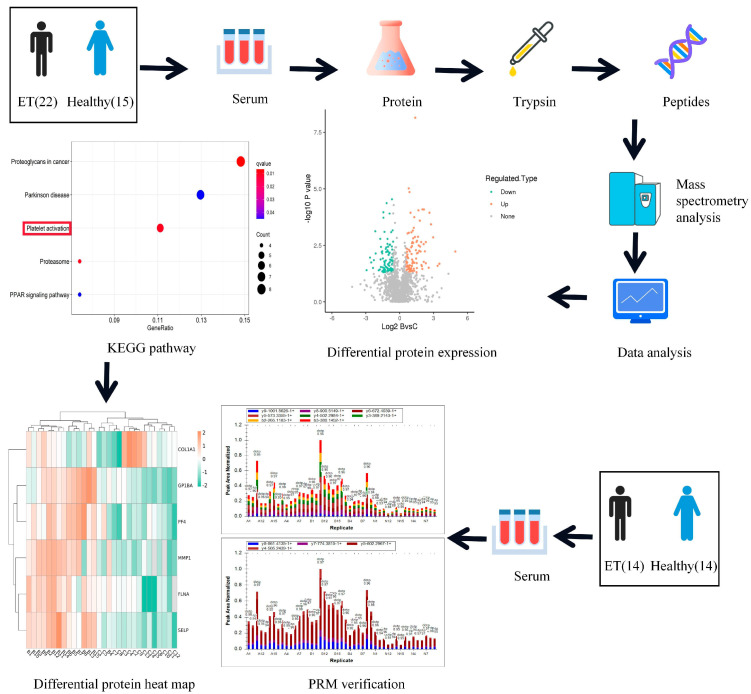
A workflow of the experiment based on serum proteomics.

A serum proteomics-based validation approach was employed to identify potential platelet activation-associated protein biomarkers in our study. Then, we employed independent validation sets to ascertain identified platelet activation-associated biomarkers using PRM. A brief description of the workflow is shown in Figure 1.

### 3.1. Quantification of Proteomic Profiling of ET and Healthy Controls

**Figure 2:**
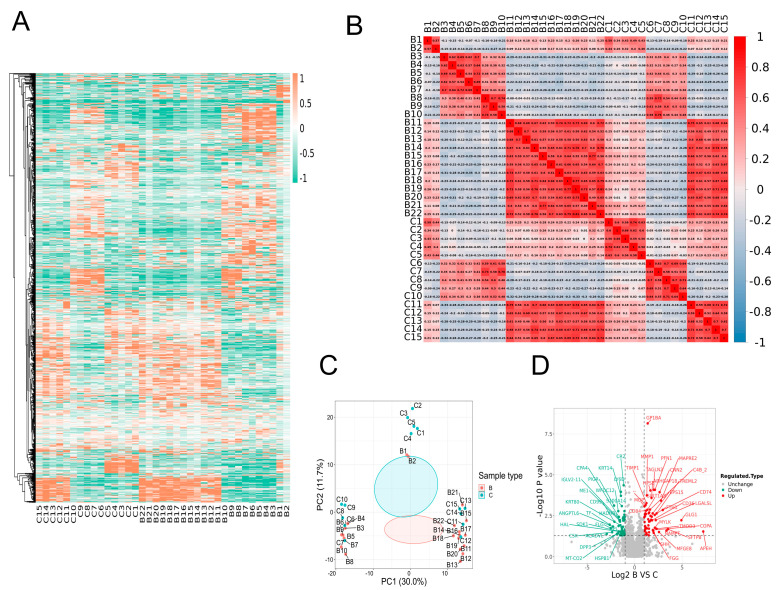
Proteomic profiling of ET patients and healthy controls. (**A**) Heatmap analysis of expression profile of proteins. (**B**) The correlation coefficient matrix of protein. (**C**) Principal component analysis in both groups. (**D**) Proteins are presented by the volcano plot. The vertical dotted lines represent proteins with a more than 1.3−fold increase (marked with red) or decrease (marked with green), respectively. The gray dots are considered as no significant change, and horizontal dotted lines display cutoff *p*−values.

We used LFQ proteomics quantitative analysis to look at the serum protein levels of 22 ET patients (B) and 15 healthy people (C) to clarify the expression of proteins related to the platelet activation pathway. We then compared and analyzed the total and differential proteins in the disease and healthy groups. The results showed that platelet activation pathway−related proteins were abnormally highly expressed in ET patients, and the specific results are as follows: During this time, a total of 1890 qualifiable proteins and 1754 quantitative proteins were found by analyzing the protein profiles of the illness group and healthy subjects (Table 1). The heatmap visualized the whole proteome comparison between B and C, which indicated a significant change (Figure 2A). The correlation coefficient matrix shows the degree of intra−sample repeatability and inter−sample correlation, and the results indicate that the experimental results are good (Figure 2B). B and C were completely separated, as shown by the principal component analysis (PCA), and each group of samples showed well−clustering based on the first two principal components (Figure 2C). A volcano plot was applied to delineate the two groups’ proteins abundance against the corresponding *p*-value obtained from the *t*−test (Figure 2D).

### 3.2. Identification of Differentially Expressed Proteins

**Figure 3:**
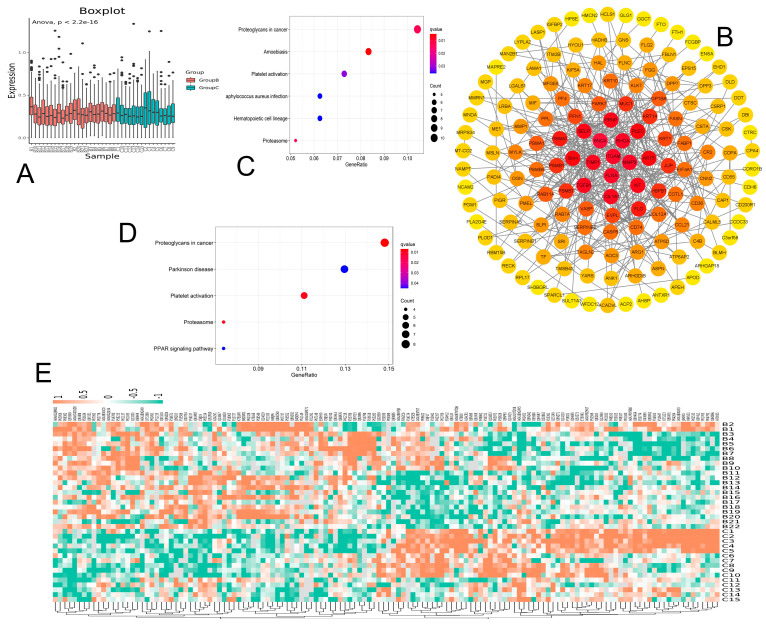
Bioinformatic analysis of differentially expressed proteins. (**A**) Boxplot of the differential proteins, with *p*−values indicating the significance of the difference between the two groups. (**B**) Differential protein PPI. The closer the protein is to the center, the more important it is in the network. (**C**) Total differential protein KEGG pathway enrichment. (**D**) Upregulated differential protein KEGG pathway enrichment. (**E**) Heatmap analysis of expression profile of differential proteins.

A total of 190 differential proteins were screened, of which 91 differential proteins had their expression downregulated, and 99 differential proteins had their expression upregulated; the comparison results are shown in a boxplot (Figure 3A). The proteins that were characterized and quantified to the differential proteins were chosen according to the change in the difference fold of 1.3 and *p* < 0.05. We entered the differential proteins into STRING to create the PPI network, and 150 nodes were produced. This allowed us to discover the interaction link between the differential proteins (Figure 3B). The results demonstrated that the platelet activation-related proteins GPIb, SELP, PF4, FLNA, COL1A1, etc., are largely concentrated in the red part of the PPI, whereas the region close to the center of the protein indicates the more relevant action values for other proteins. We conducted a KEGG pathway enrichment analysis of differential proteins to determine the pertinent pathways that may be activated. The results revealed a total of six pathways with differential expression; primarily, proteoglycans in cancer, amoebiasis, platelet activation, Staphylococcus aureus infection, hematopoietic cell lineage, and proteasome are just a few of the pathways that were enriched for upregulated protein pathways (Figure 3C); the other four are PPAR signaling and proteoglycans in cancer, Parkinson’s disease, platelet activation, and proteasome (Figure 3D). The results suggest that the platelet activation pathway was significantly activated, and the GPIbα, SELP, PF4, MMP1, FLNA, and COL1A1 were the differentiating proteins linked to the platelet activation pathway. The heatmap visualizes the differentially expressed protein comparison between B and C, which indicated a significant change (Figure 3E).

### 3.3. Validation of Platelet Activation Protein Results

To further validate the LFQ proteomics results, we quantified the above proteins usng PRM technology. In total, 14 ET patients (ET group) and 14 healthy people (healthy group) were randomly selected for their first visit to our institution. As some patients were not newly diagnosed patients, they had started treatment at the time of their first visit to our institution, and their treatment regimens were different, as detailed in Supplementary Table S1. The results show that the serum GPIbα, SELP, PF4, MMP1, and FLNA protein levels were significantly lower than those in healthy subjects, with significant differences (*p* < 0.0001), and the COL1A1 levels were lower in ET patients than in healthy controls, although there were no statistically significant changes (see Table 2 for particular results). This suggests that platelet activation pathway-related protein expression is upregulated in ET patients.

### 3.4. Serum SELP Levels Are Related to the Prognosis of ET Patients

#### 3.4.1. Positive Correlation between Serum SELP Levels and Prognostic Scores

**Figure 4:**
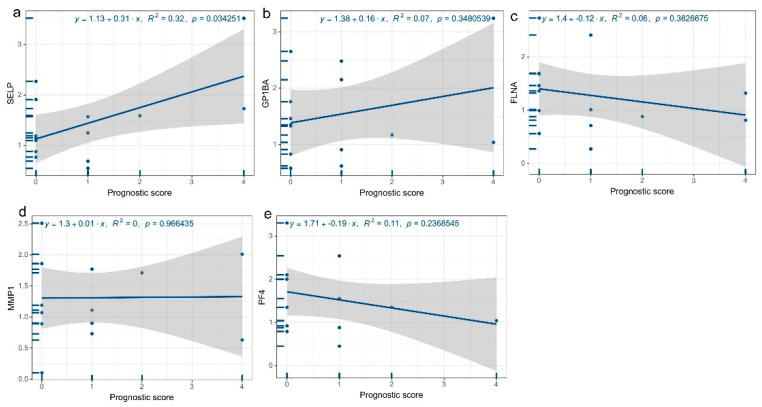
Regression plots of platelet activation-related proteins and prognostic scores. (**a**) SELP vs. prognostic score regression, (**b**) GPIbα vs. regression of the prognostic score, (**c**) FLNA vs. prognostic score regression, (**d**) MMP1 vs. prognostic score regression, (**e**) PF4 vs. prognostic score regression.

According to the aforementioned findings, there were significantly higher serum levels of GPIbα, SELP, PF4, MMP1, FLNA, and other proteins than in healthy subjects. To further understand whether the above proteins were related to the prognostic scores of ET patients, in the aforementioned 14 patients, we conducted a regression analysis to determine the expression levels of serum platelet activation-related proteins and prognosis scores. The results reveal a substantial positive correlation between serum SELP levels and prognostic scores (R^2^ = 0.32, *p* = 0.034) (Figure 4a), while GPIbα (R^2^ = 0.07, *p* = 0.348) (Figure 4b), FLNA (R^2^ = 0.06, *p* = 0.383) (Figure 4c), MMP1 (R^2^ = 0.11, *p* = 0.237) (Figure 4d), and PF4 (R^2^ = 0, *p* = 0.966) (Figure 4e) were not significantly correlated with prognostic scores despite being all linked.

#### 3.4.2. Serum SELP Levels Are Highly Expressed in ET Patients with Prognostic Risk Factors (Including Advanced Age, Leucocytosis, and History of Thrombosis)

**Figure 5:**
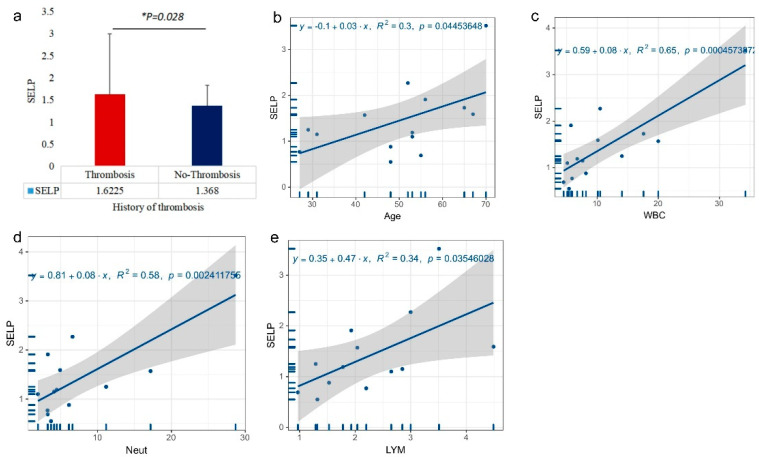
Plots of prognostic risk factors and SELP expression. (**a**) Difference in SELP expression between patients with and without a history of thrombosis, (**b**) regression of age and SELP levels, (**c**) regression of white blood cell count and SELP levels, (**d**) regression of neutrophil count and SELP levels, (**e**) regression of lymphocyte count and SELP levels.

We examined the link between each prognosis risk factor and SELP levels separately to better understand the connection between SELP levels and prognostic risk factors (history of thrombosis, age, and white blood cell count). First, we split the above 14 ET patients into two groups: patients with a history of thrombosis (4 patients) and patients without a history of thrombosis (10 patients) and examined the levels of serum SELP expression in the two groups. There were significant differences between the two groups (Figure 5a). Then, to clarify the correlation between serum SELP expression levels and age and white blood cell count in ET patients, we conducted a regression analysis of age, serum SELP expression levels, and white blood cell count (including neutrophil count, lymphocyte count, and neutrophil/lymphocyte count ratio), and the results reveal that age (R^2^ = 0.3, *p* = 0.045) (Figure 5b), white blood cell count (R^2^ = 0.65, *p* = 0.000) (Figure 5c), neutrophil count (R^2^ = 0.58, *p* = 0.002) (Figure 5d), and lymphocyte count were positively connected with patients’ serum SELP levels (*p* < 0.05) (Figure 5e, R^2^ = 0.34, *p* = 0.035).

### 3.5. Serum SELP Levels Are Associated with Coagulation Abnormalities

#### 3.5.1. Serum SELP Levels Were Negatively Correlated with Coagulation Factors AT-III and Fbg

**Figure 6:**
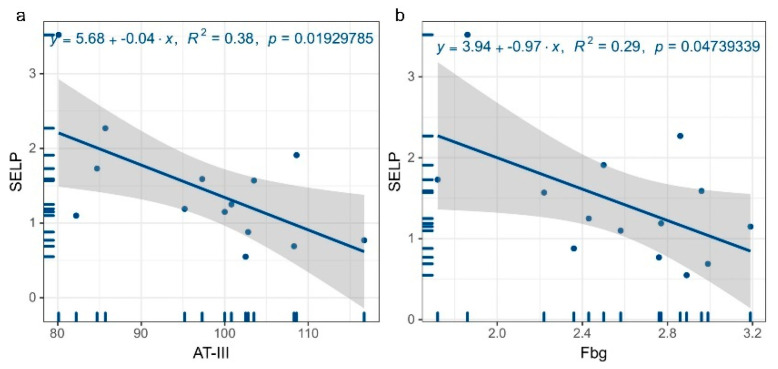
Regression plots of SELP and coagulation factor levels. (**a**) Regression plot of SELP and AT-III levels, (**b**) regression plot of SELP and Fbg levels.

The above results indicate that SELP levels differ significantly between patients with and without a history of thrombosis. To further assess the effect of serum SELP expression on coagulation function, we performed a regression analysis of the expression levels of coagulation full indexes, thromboelastography indexes, and serum SELP levels. The coagulation full indexes included plasma D-dimer assay (D-Dimer), prothrombin time, prothrombin activity (PTA), prothrombin time ratio (INR), activated partial thromboplastin time, thromboplastin time, fibrinogen concentration (Fbg), fibrin degradation product, and antithrombin III (AT-III); the thromboelastography index included coagulation time (R), coagulation rate (K), fibrin function (Angle), and platelet aggregation (MA). The results show that serum AT-III (R^2^ = 0.38, *p* = 0.019) (Figure 6a) and Fbg (R^2^ = 0.29, *p* = 0.047) (Figure 6b) were inversely connected with SELP levels (*p* < 0.05). (Other results are shown in Supplementary Figure S1.)

#### 3.5.2. Lower AT-III and Higher Fbg in Patients with a History of Thrombosis

**Figure 7:**
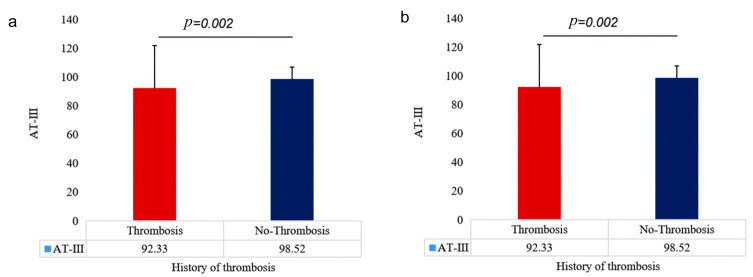
Differences in coagulation factor levels between patients with and without a history of thrombosis. (**a**) AT-III expression varies across patients with and without a history of thrombosis, (**b**) differences in Fbg expression in patients with and without thrombotic history.

We further divided 60 ET patients recruited from outpatient clinics into two groups based on their history of thrombosis (11 cases) and their lack of history of thrombosis (49 cases), and we compared the serum AT-III and Fbg expression levels. This helped us better understand the relationship between AT-III and Fbg and thrombosis. AT-III levels were significantly lower in patients with a history of thrombosis compared with those without a history of thrombosis (92.33 vs. 98.52, *p* = 0.002) (Figure 7a), while there were statistically significant differences in the results; the Fbg levels in patients with a history of thrombosis were significantly higher than those in patients without a history of thrombosis (2.86 vs. 2.52, *p* = 0.013) (Figure 7b).

### 3.6. Serum SELP Promotes Coagulation Abnormalities via Inflammatory Factors

#### 3.6.1. Inflammatory Factor Expression May Lead to Coagulation Abnormalities

**Figure 8:**
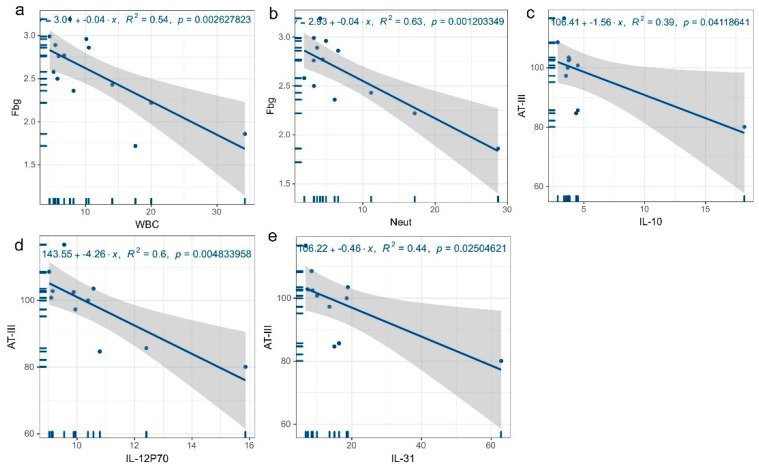
Regression plots of SELP-related indicators and coagulation factor levels. (**a**) Leukocyte count versus Fbg level, (**b**) neutrophil count concerning Fbg level, (**c**) IL-10 concerning AT-III level, (**d**) IL-12P70 concerning AT-III level, (**e**) IL-31 concerning AT-III level.

According to the results above, thrombosis is associated with decreased AT-III, increased Fbg, and SELP levels are adversely correlated with both AT-III and elevated Fbg. To understand the mechanism by which SELP affects AT-III and Fbg levels, we further correlated age, white blood cell count (including neutrophils and lymphocytes), IL-1beta, IL-1a, IL-4, IL-6, IL-8, IL-10, IL-12P70, IL-13, IL-17A, IL-27, IL-31, IL-33, IFNr, MIP-1a, MIP-1beta, TNFa, IP-10, MCP-1, VEGF-D, and VEGF-A with AT-III and Fbg using regression analysis, and the findings reveal that IL-10 (R^2^ = 0.39, *p* = 0.041) (Figure 8c), IL-12P70 (R^2^ = 0.6, *p* = 0.005) (Figure 8d), and IL-31 (R^2^ = 0.44, *p* = 0.025) (Figure 8e) were inversely connected with AT-III levels. Both the white blood cell count (R^2^ = 0.54, *p* = 0.003) and the neutrophil count (R^2^ = 0.63, *p* = 0.001) showed statistically significant negative correlations with Fbg levels, as shown in Figure 8a and Figure 8b, respectively. (Other results are shown in Supplementary Figure S2.)

#### 3.6.2. Serum SELP Promotes the Expression of Inflammatory Factors

**Figure 9:**
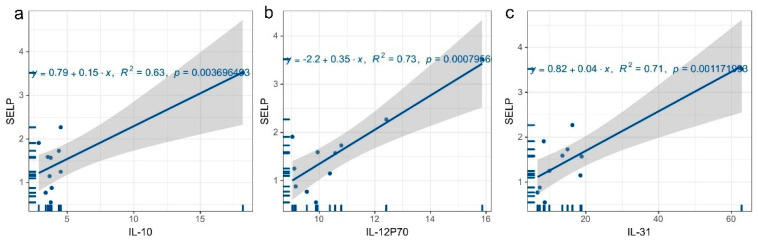
Regression plots of SELP and inflammatory factor levels. (**a**) Regression plots of SELP and IL-10 levels, (**b**) regression plots of SELP and IL-12P70 levels, (**c**) regression plots of SELP and IL-31 levels.

The above results show that serum SELP levels were positively correlated with the leukocyte, neutrophil, and lymphocyte counts. We conducted regression analysis on the expression levels of the aforementioned inflammatory factors (such as IL-10, IL-12P70, and IL-31) and SELP levels to better comprehend the link between serum SELP levels and inflammatory factors. The findings demonstrated a positive correlation between serum inflammatory factors levels and SELP levels (*p* < 0.01) for IL-10 (R^2^ = 0.63, *p* = 0.004), IL-12P70 (R^2^ = 0.73, *p =* 0.000) (Figure 9b), and IL-31 (R^2^ = 0.71, *p* = 0.001) (Figure 9c). (Other results are shown in Supplementary Figure S3.)

## 4. Discussion

Elevated primary platelet counts and a higher likelihood of thrombosis are the key characteristics of the clinical symptoms of ET. Studies in the literature have shown that the incidence of thrombosis in ET patients at diagnosis is about 20.7% [16], the incidence of thrombosis during follow up is about 12% [1], and the majority of thrombotic sites are in the heart and brain, and the occurrence of thrombotic events significantly lowers patient survival and quality of survival.

Platelet activation has a key role in thrombogenesis and thromboinflammatory activation, and the rate of platelet activation is significantly higher in patients with ET [8]. By using LFQ proteomics, this study discovered that the platelet activation pathway was abnormally activated in ET patients. Platelet activation-related proteins have a key role in the occurrence of platelet activation. The results of the current investigation via LFQ and PRM tests were compatible with the previous studies. Among them, platelet activation-related proteins, such as GPIbα [17], SELP, PF4 [18], and MMP1 [19], were reported to be enhanced in ET. Meanwhile, the present study found for the first time that the platelet activation-related protein FLNA [20] was elevated in ET patients, further demonstrating the abnormal activation of the platelet activation pathway in ET patients. FLNA is required for the spreading of activated platelets and plays an important role in maintaining the normal structure and size of platelets [21]. FLNa has also been shown to be required for GPIbα trafficking, and GPIb-IX reached the cell surface only when coexpressed with FLNa. GPIb-IX remained in the cytoplasm in the absence of FLNa [22]. Previous studies have shown that platelet count is related to FLNA expression level [23], but most studies have not found a direct link between FLNA and thrombosis, which may also be the reason why the FLNA level was not found to be significantly correlated with risk score in this study.

The association between serum SELP and IPSET prognostic scores and prognostic risk variables (such as thrombotic history, age, and leukocyte count) in ET patients is being examined for the first time in this study. SELP belongs to the selectin family of proteins and is mainly expressed by platelets, endothelial cells, and immune cells. In the mechanism of thrombus formation through activated platelets, SELP plays a significant role [24]. A positive correlation between age and SELP expression has been found in diseases related to hyperlipidemia and thrombosis [24,25], and the same was found in the present study in ET patients. It has long been known that leukocytes and SELP interact, with SELP expressed on active platelet membranes and endothelial cells interacting with (PSGL-1), the major cell adhesion partner of leukocytes [26], to mediate leukocyte chemotaxis and encourage inflammatory pathways [27,28,29]. Our discovery that peripheral blood leukocyte counts, age, and a history of thrombosis were all related to serum SELP levels in ET patients further supports these findings and suggests a possible mechanism by which serum SELP levels are related to prognosis in ET patients.

SELP is a key protein for platelet activation and plays an important role in coagulation function [30]. In conditions such as atherosclerosis [31,32,33], where AT-III, Fbg, and SELP interact, several studies have shown that SELP is negatively connected with the levels of several coagulation factors such as AT-III [34] and Fbg [35]. AT-III is currently used as a prognostic [36] and efficacy criterion in a variety of thrombosis-related diseases [37,38]. AT-III supplementation is frequently used in the treatment and control of critically ill patients in a variety of diseases [39] because it not only has an anticoagulant effect but also inhibits the inflammatory response [36], which is important for the prognosis of a variety of diseases [40]. The results of this study show that AT-III was significantly lower in ET patients with a history of thrombosis, further suggesting the role of AT-III in the anticoagulation in ET patients.

Inflammatory stimuli can trigger the coagulation cascade response, which is now a widely acknowledged theory about the relationship between inflammation and coagulation [41]. This view is further supported by the present study showing a strong association between leukocytes, inflammatory factors, and coagulation factors (AT-III, Fbg) in patients with ET. It has been demonstrated that several inflammatory substances, including IL-10 and IL-12, can lower the levels of several coagulation-related substances, including AT-III.

In this study, we discovered a favorable correlation between leukocyte counts and the expression of several inflammatory conditions, and serum SELP levels in ET patients. These findings have significant implications for the promotion of the inflammatory status of ET patients. In addition, consistent with the findings in the literature, we found that SELP promoted the expression of the inflammatory factors IL-10 [42] and IL-12 [43,44] in the sera of ET patients. In this study, we also discovered for the first time a positive correlation between serum SELP expression and IL-31 in ET patients. Previous studies have shown that IL-31 can increase the levels of TXA2 and TXB2. At the same time [45], IL-31 can promote the occurrence of cardiovascular diseases [46,47], participates in a variety of hematological malignancies and the occurrence of pruritus, and is positively correlated with the level of pruritus [48]. It not only participates in the occurrence of a variety of inflammatory processes but also plays an important role in vascular endothelial injury and thrombosis [49]. The finding of an inverse correlation between IL-31 levels and AT-III levels in the present study also further suggests that IL-31 may be involved in thrombosis. We hypothesized that SELP may contribute to abnormal coagulation by encouraging the upregulation of the inflammatory factors IL-10, IL-12, and IL-31 and inhibiting the expression of coagulation factor AT-III.

In conclusion, SELP is one of the most crucial proteins for platelet activation. We think that there is considerable platelet activation in ET patients compared to healthy participants, which not only participates in the adhesion and aggregation of platelets to endothelial cells, increasing thrombosis and encouraging coagulation, but also binds to leukocytes, mediates thrombosis, stimulates leukocytes, and encourages the release of a variety of inflammatory substances, which, in turn, leads to a decrease in AT-III and inhibits anticoagulation. This leads to a decrease in AT-III, which inhibits anticoagulation and leads to thrombosis, thus affecting the prognosis of ET patients. We conclude that serum SELP should be promoted and further investigated in the clinical setting as it may be a quick and accurate indicator to determine the prognosis of ET patients.

## 5. Limitations

As this study is not a rigorous RCT study, and there are few cases included in the study, the results may be biased to some extent. At the same time, as this study is a clinical study, the relationship between SELP and various factors and thrombosis is only a speculative result, which needs to be confirmed by further laboratory studies.

## Figures and Tables

**Table 1:** Overview of protein identification.

Title	Number
Total spectrums	2,833,072
Matched spectrums	610,071
Peptides	13,951
Unique peptides	12,376
Identified proteins	1890
Quantifiable proteins	1754

**Table 2:** Proteomic verification of differential proteins related to platelet activation signaling pathway.

Protein	Relative Protein Abundance		
	Health Group	ET Group	*p* (Health vs. ET Group)
GPIbα	0.52 ± 0.19	1.53 ± 0.78	0.0000
SELP	0.41 ± 0.12	1.49 ± 0.77	0.0000
PF4	0.58 ± 0.34	1.54 ± 0.76	0.0000
MMP1	0.17 ± 0.09	1.39 ± 0.71	0.0000
FLNA	0.39 ± 0.24	1.41 ± 0.83	0.0001
COL1A1	1.13 ± 0.54	0.98 ± 0.22	0.6670

## Data Availability

The original contributions presented in the study are included in the article/Supplementary Material. Further inquiries can be directed to the corresponding author.

## Supplementary Materials

The following supporting information can be downloaded at: https://www.mdpi.com/article/10.3390/jcm12031078/s1, Table S1: Basic information of 14 patients; Figure S1: Regression analysis of coagulation factor and SELP; Figure S2: Regression analysis of inflammatory factors and coagulation factors; Figure S3: Regression analysis of inflammatory factors and SELP.

## Abbreviations

ET	essential thrombocythemia
LFQ	label-free quantification
PRM	parallel reaction monitoring
IPSET	International Prognostic Score for ET
AT-III	antithrombin
GO	gene ontology
KEGG	Kyoto Encyclopedia of Genes and Genomes
PCA	principal component analysis
Fbg	fibrinogen concentration
