# Supplementary material for: Proteomic-Based Platelet Activation-Associated Protein SELP May Be a Novel Biomarker for Coagulation and Prognostic in Essential Thrombocythemia
Source: J Clin Med. 2023 Jan 30;12(3):1078. doi: 10.3390/jcm12031078 (PMC9917633; doi:10.3390/jcm12031078)
Supplement: Supplementary file 1 [file jcm-12-01078-s001.zip › jcm-2074124-supplementary.pdf]

Supplementary Materials

Table S1. Basic information of 14 patients

patients	1	2	3	4	5	6	7	8	9	10	11	12	13	14
Age	27	42	31	29	48	53	56	70	53	52	65	67	48	55
gender	male	male	male	male	male	male	female	female	male	female	male	male	male	female
hydroxyurea		yes				yes		yes	yes		yes	yes	yes	
interferon	yes					yes	yes		yes	yes				
thrombosis								stroke			AMI		AMI	TIA
aspirin						0.1/d	0.1/d	0.1/d	0.1/d	0.1/d	0.1/d	0.1/d	0.1/d	0.1/d
PLT before treatment	528	511	494	462	532	526	778	654	508	471	680	622	467	590
PLT after treatment	440	327	522	459	529	467	539	227	303	460	145	514	433	486
WBC before treatment	5.91	19.99	7.63	14.06	8.18	6.75	5.73	34.23	5.14	10.5	17.59	10.12	5.41	4.54
WBC after treatment	5.46	6.01	7.52	5.97	7.88	5.05	3.38	6.61	8.5	6.06	9.49	7.37	5.41	4.06
Neutrophil before treatment	3.22	17.17	4.1	11.15	6.12	4.47	3.26	28.69	1.92	6.6	13.8	4.92	3.69	3.24
Neutrophil after treatment	3.04	4.2	4.09	3.86	5.96	3.62	1.45	3.99	4.39	3.49	7.65	4.08	3.69	2.42
Lymphocyte before treatment	2.2	2.04	2.85	1.29	1.53	1.78	1.93	3.51	2.65	3		4.49	1.32	0.97
Lymphocyte after treatment	1.97	1.35	2.84	1.65	1.43	1.11	1.6	2.09	3.32	1.79	1.21	3.01	1.32	1.1
SELP before treatment	0.77	1.57	1.15	1.25	0.88	1.19	1.91	3.52	1.1	2.27	1.73	1.59	0.55	0.69
SELP after treatment	0.69	1.59	0.96	1.09	0.92	1.34	0.72	2.55	1.15	1.34	0.66	1.28	0.63	1.27
AT-III before treatment	116.7	103.5	100	100.8	102.8	95.2	108.6	80.1	82.2	85.7	84.7	97.3	102.5	108.3
AT-III after treatment	102.1	101.2	100.4	95.9	107	96.1	114.6	76.1	88.2	88.8	86	84	99.7	98.4
Fbg before treatment	2.76	2.22	3.19	2.43	2.36	2.77	2.5	1.86	2.58	2.86	1.72	2.96	2.89	2.99
Fbg after treatment	2.61	2.49	2.68	2.34	2.91	2.59	3.41	1.54	2.28	3.04	1.95	2.57	4.36	2.68

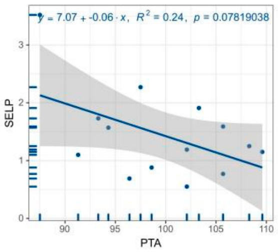

a

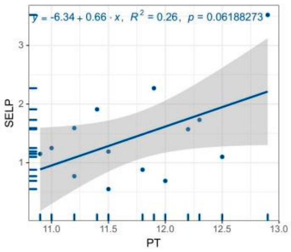

b

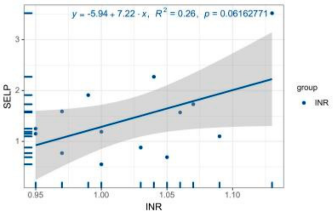

c

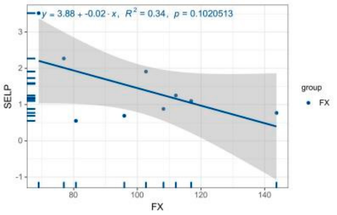

d

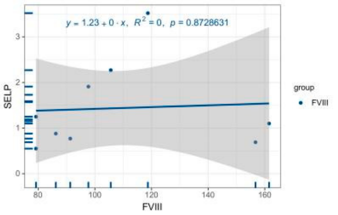

e

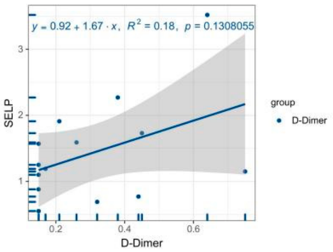

f

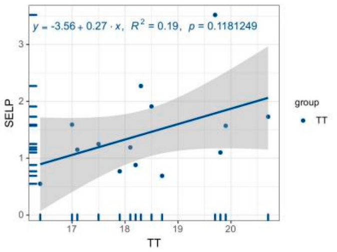

g

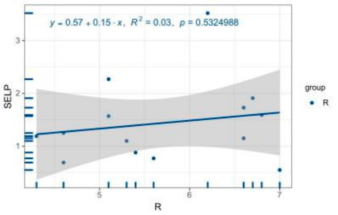

h

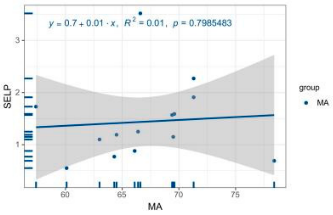

i

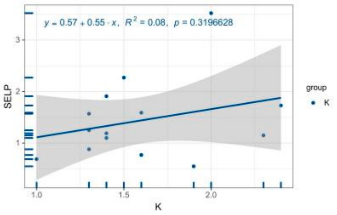

j

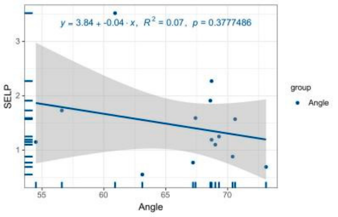

k

a. Regression analysis of PTA and SELP, b. Regression analysis of PT and SELP, c. Regression analysis of INR and SELP, d. Regression analysis of FX and SELP, e. Regression analysis of FⅧ and SELP, f. Regression analysis of D-Dimer PTA and SELP, g. Regression

analysis of TT and SELP, h. Regression analysis of R and SELP, i. Regression analysis of MA and SELP, j. Regression analysis of K and SELP, k. Regression analysis of Angle and SELP.

Figure S1. Regression analysis of coagulation factor and SELP

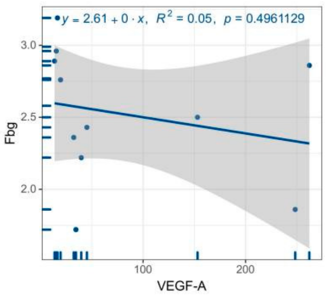

a. Regression analysis of VEGF-A and Fbg

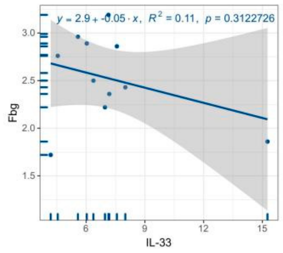

b. Regression analysis of IL-33 and Fbg

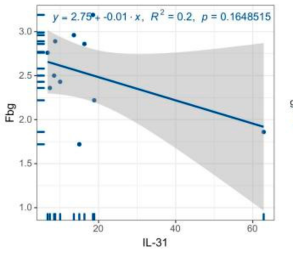

c. Regression analysis of IL-31 and Fbg

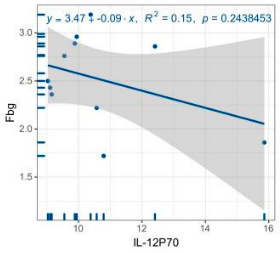

d.Regression analysis of IL-12P70 and Fbg

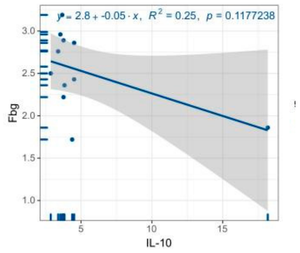

e. Regression analysis of IL-10 and Fbg

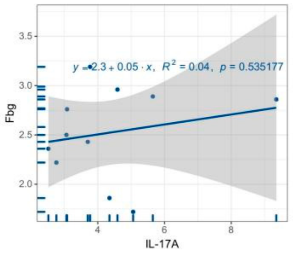

f. Regression analysis of IL-17A and Fbg

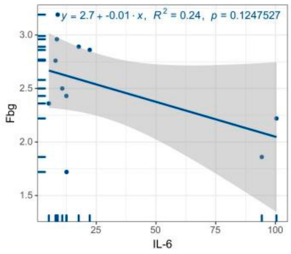

g. Regression analysis of IL-6 and Fbg

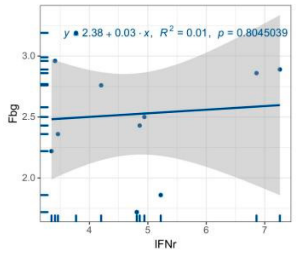

h. Regression analysis of IFN-r and Fbg

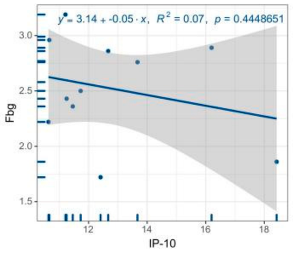

i. Regression analysis of IP-10 and Fbg

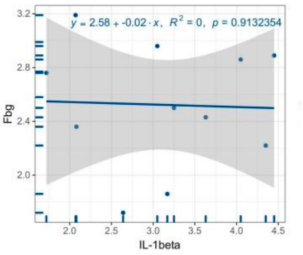

j. Regression analysis of IL-1beta and Fbg

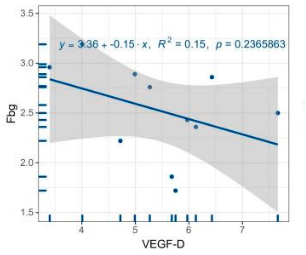

k. Regression analysis of VEGF-D and Fbg

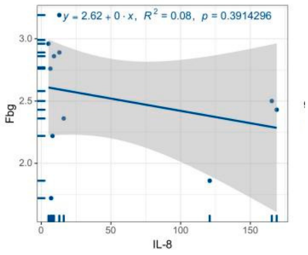

l. Regression analysis of IL-8 and Fbg

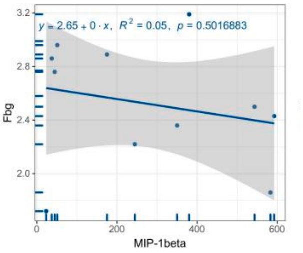

m. Regression analysis of MIP-1beta and Fbg

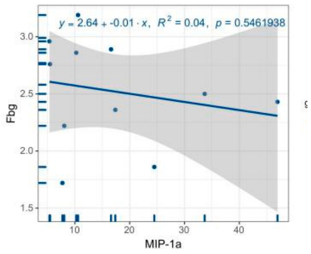

n. Regression analysis of MIP-1a and Fbg

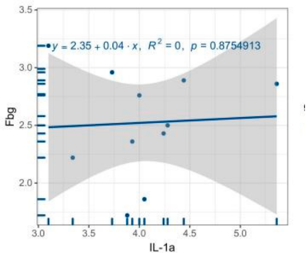

o. Regression analysis of IL-1a and Fbg

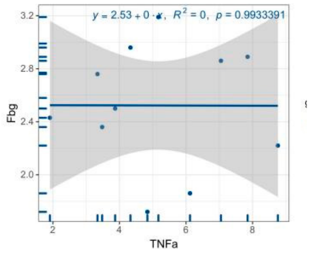

p. Regression analysis of TNFa and Fbg

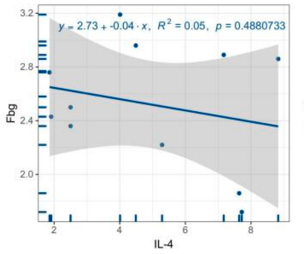

q. Regression analysis of IL-4 and Fbg

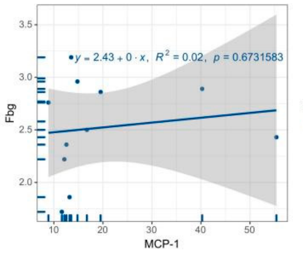

r. Regression analysis of MCP-1 and Fbg

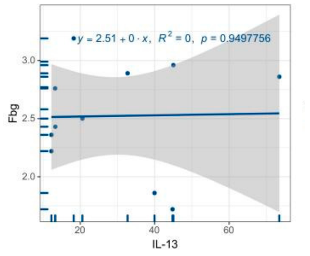

s. Regression analysis of IL-13 and Fbg

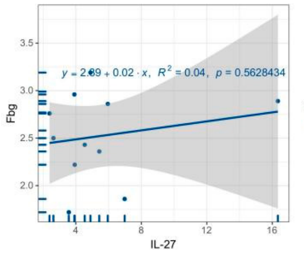

t. Regression analysis of IL-27 and Fbg

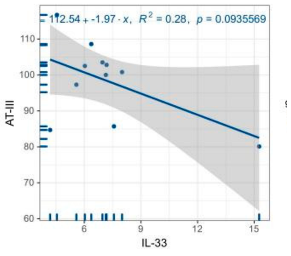

u. Regression analysis of IL-33 and AT-III

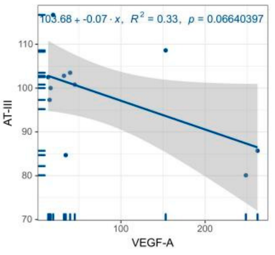

v. Regression analysis of VEGF-A and AT-III

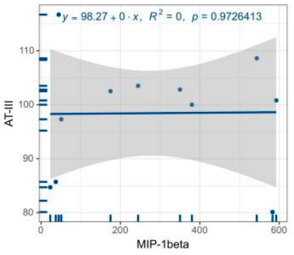

w. Regression analysis of MIP-1beta and AT-III

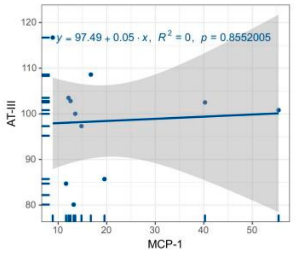

x. Regression analysis of MCP-1 and AT-III

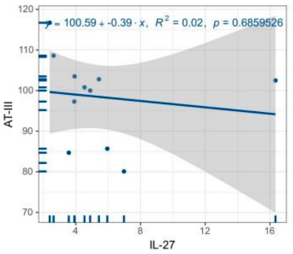

y. Regression analysis of IL-27 and AT-III

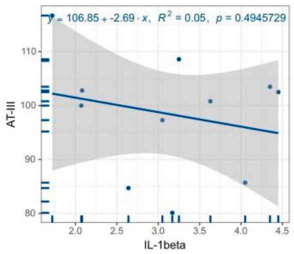

z. Regression analysis of IL-1beta and AT-III

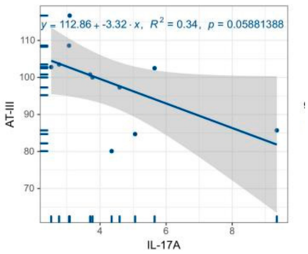

aa. Regression analysis of IL-17A and AT-III

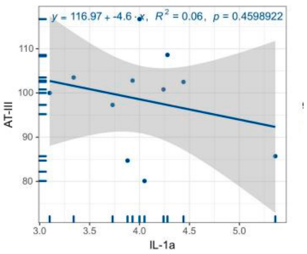

ab. Regression analysis of IL-1a and AT-III

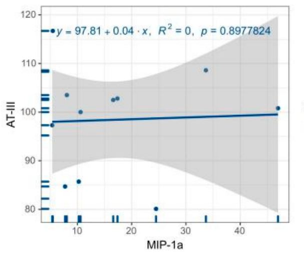

ac. Regression analysis of MIP-1a and AT-III

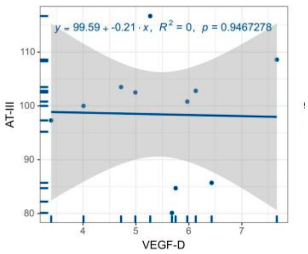

ad. Regression analysis of VEGF-D and AT-III

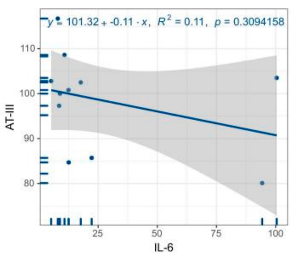

ae. Regression analysis of IL-6 and AT-III

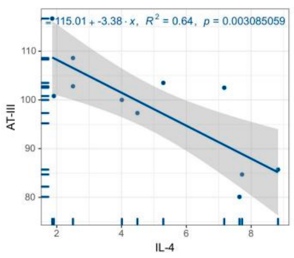

af. Regression analysis of IL-4 and AT-III

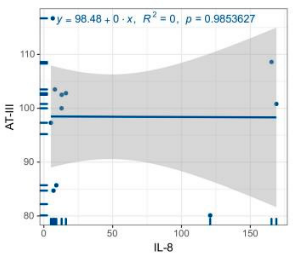

ag. Regression analysis of IL-8 and AT-III

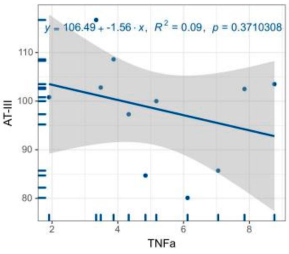

ah. Regression analysis of TNFa and AT-III

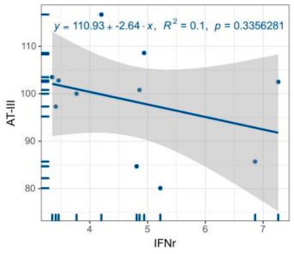

ai. Regression analysis of IFNr and AT-III

Figure S2. Regression analysis of inflammatory factors and coagulation factors

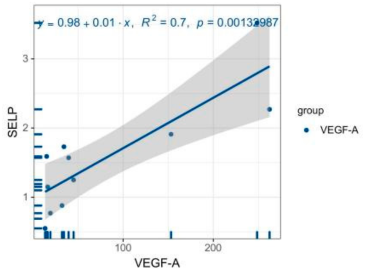

a

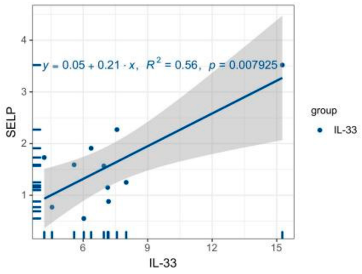

b

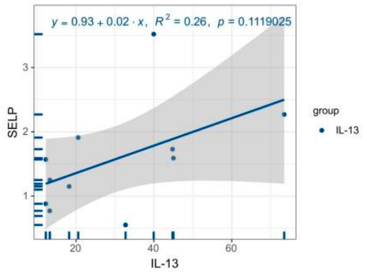

c

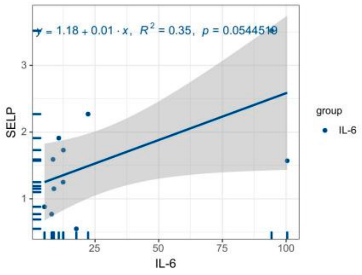

d

a. Regression analysis of VEGF-A and SELP, b. Regression analysis of IL-33 and SELP, c. Regression analysis of IL-13 and SELP, d. Regression analysis of IL-6 and SELP.

Figure S3. Regression analysis of inflammatory factors and SELP
